# Skin tumor identification by means of convolutional neural network and improved gray wolf optimizer

**DOI:** 10.3389/fonc.2026.1724577

**Published:** 2026-05-05

**Authors:** Mei Ou, Hongmei Wu, Hong Liu, Jing Zhu

**Affiliations:** 1 Department of Dermatology, Hamilton Medical Aesthetic Hospital, Chengdu, Sichuan, China; 2 Department of Dermatology, The Second People’s Hospital of Neijiang, Neijiang, Sichuan, China; 3 Department of Dermatology, Zizhong People’s Hospital, Neijiang, Sichuan, China; 4 Department of Radiology, The General Hospital of Western Theater Command, Chengdu, Sichuan, China

**Keywords:** convolutional neural network, deep learning, improved gray wolf optimization algorithm, skin, skin cancer diagnosis

## Abstract

**Introduction:**

Early and accurate diagnosis of skin cancer has a huge impact on the survival rate of patients and deep learning-based CNN and dermatologists' intelligence support can fasten the diagnosis not only within the clinics but also outside.

**Methods:**

This paper introduces a hybrid classification framework for automatic detection of melanoma that combines deep learning and better optimization. A modified GWO method called improved GWO (IGWO) has been proposed which can efficiently optimize CNN parameters and feature learning. Conventional GWO has drawbacks such as early convergence and poor exploration in high-dimensional spaces, so the IGWO regenerates the weak omega agents based on their fitness level. The underperforming omega wolves were discarded in every step and then either the elite solutions (alpha, beta, delta) or the stochastically resampled solution was put to the wolves so that both exploration and exploitation would reach better state.

**Results:**

The presented CNN/IGWO model has been experimented with a skin cancer dataset SIIM-ISIC 2020. The proposed model yielded test accuracy of 98.47% and AUC of 98.2 respectively that were both higher than those models using basic GWO method and other state-of-the-art deep learning methods.

**Discussion:**

These outcomes show that using the IGWO mechanism along with training of CNN speeds up convergence, enhances the solution and thus the classification performance. The proposed method shows that intelligence-based optimization could give more practical, accurate results in automated melanoma diagnosis.

## 1 Introduction

Each of our bodies is made up of intricately organized parts that are arranged in harmony with one another. We will feel healthy because of this order and harmony, but anytime there is even a slight disruption in this intricate system, our body’s general balance is upset, and we become ill (1). Our bodies are continually at risk from little and large illnesses (2). Cancer is among the most prevalent and distinctive diseases (3). No area of the body is immune to cancer, given the many forms it can take. In this post, we’ve spoken about the numerous characteristics of skin cancer, one of the cancer forms.

The largest organ in the human body is the skin (4). This covering organ does its best to protect all the internal organs of our body from all external factors (5). The skin is exposed to sunlight, heat, infection, injuries caused by cuts, burns and bruises more than other organs. For this reason, there is a possibility of the skin contracting various diseases (6).

The skin is also a place to store water, fat and vitamin D. The skin consists of two layers: an outer layer visible to the eye and an inner layer that is not. This top layer is always exposed to all kinds of damage (7).

Skin cancer is a serious condition that can cause irreparable damage to the skin. Sometimes the skin of the body undergoes changes in its tissue and malignant cells are formed in the skin tissue. These cells can cause cancer in this part of the body (8).

Depending on the cause and cell type involved, skin cancer is divided into different categories. Each of these types has a different severity and may occur at different ages (9). The most well-known type of skin cancer is melanoma (10).

Our skin is made up of pigments called melanin. Sometimes on the skin of some of us, moles with an unusual shape are created, which are actually places where melanin accumulates. These moles can be signs of melanoma, especially if they appear in adulthood. In men, moles are located on the chest and back, and for women, they are more visible on the legs. If this cancer is not detected and treated in time, it may spread throughout the body and then it will be very difficult to treat. This type of skin cancer is much more dangerous than other types and it kills many people every year (11).

Most of the skin cancers we diagnose are not detected by patients during personal skin exams. This means that while self-examination is a very important tool in the diagnosis and treatment of skin cancer, it should not replace a professional checkup by a trained skin cancer doctor (12).

In cases of cancer, early detection and appropriate treatment improve recovery and survival rates and survival of patients (13). Therefore, even though cancer diagnosis relies on interventional techniques like surgery, radiation treatment, and chemotherapy, studies indicate that the application of contemporary technological tools like image analysis techniques in related processes has been successful in identifying and categorizing tumors (14).

Image segmentation is useful to clinicians as a decision-making tool for the early identification of tumors. Early detection of cancer through screening images has the most important contribution in reducing mortality from a specific cancer (15). Medical imaging plays an important role in all stages of prediction, screening, identification, staging, treatment planning, response evaluation, recurrence, and palliation of cancer. In other words, images form an important part of cancer clinical protocols and have the ability to provide functional, metabolic, structural and morphological information and help in clinical decision-making along with other diagnostic tools (16).

The science of image processing has made remarkable progress in both theoretical and practical aspects in the last few decades. Image correction, performance improvement, and form modification can all be achieved within the practical and technological framework of image analysis and evaluation. It includes information compression, image enhancement, and pattern recognition, and it is also the output stage, which can be an image or a report obtained from the result of image analysis. In this sense, there have been several attempts in this area in recent years. The following research findings might be highlighted.

Xu et al. conducted research on a soft−computing−based machine approach for diagnosing skin tumors. A method for the automated computer-assisted early detection of skin cancer is provided. Malignant cells develop in the skin tissues in a condition known as skin cancer. Early diagnosis of this illness aids in its treatment by the therapists. Ten alternative methodologies from the literature have been evaluated using simulations of the American Cancer Society database. Accuracy, sensitivity, negative forecasting value, validity, and positive forecasting amount were all higher for this procedure than for earlier ones. Pérez et al. (17) conducted a thorough experimental analysis using CNN method to automatically diagnose cancer. Even for experienced dermatologists, diagnosing melanoma can be challenging since there are many different morphologies seen in patients’ moles. 5 stages made up a research project that examined how sensitive the systems were to the optimization strategy that was employed for training them. Significant suggestions were also included, making it simpler to choose the right convolutional model and approach based on the information’s features.

Pérez et al. (18) examined into a genetic algorithm-driven ensemble-based CNN approach for skin cancer detection. Melanoma is one of the primary causes of tumor-related death. Early diagnosis and much lower mortality can be achieved through the development of soft computing technologies. In this paper, we present an ensemble learning− and genetic algorithm−inspired convolutional neural network architecture for skin lesion detection. This work provided more evidence that genetic algorithms may be successfully used to identify appropriate structures for the detection of malignant tumors, with overall simulation results in dermoscopic and non-dermoscopic pictures outperforming the nearer modeling by 11% and 13%, respectively.

Efimenko et al. (19) acknowledged the application of neural network models in medical imaging technology for identifying skin cancers. One of the most dangerous cancers, melanoma has become a major global issue. Malignant melanoma is estimated to cause 66,000 fatalities and 132,000 new cases globally each year, according to the World Health Organization. Neural networks outperform dermatologists in terms of specificity, precision, and sensitivity, according to the articles evaluated. Neural networks can assess aspects that the human eye may not be able to see. Despite this, we still require further datasets to support those claims. These days, computer vision is becoming a useful technique for early skin diagnosis of diseases, particularly skin cancer.

Kaur et al. (20) identified melanoma using a novel deep convolutional neural network applied to manually segmented images. The problem of melanoma identification using dermoscopic skin samples is quite difficult. This study suggests a CNN-based automatic melanoma diagnosis. Comparing the suggested DCNN classifier to other cutting-edge networks, it performed well. On the ISIC 2016, 2017, and 2020 datasets, the proposed DCNN classifier performed exceptionally well, outperforming existing cutting-edge techniques. This suggested strategy could offer a simpler yet more sophisticated platform for automating the skin cancer diagnosis process.

In this work, medical image pictures of melanoma disease and a pre-processing technique are used to improve the image accuracy (then extracting mesh areas and combining features and limiting their further processing based on texture and shape features and reducing dimensions based on LDA analysis of linear discriminant model, which to discovery the finest features among the features that we have extracted, we have used an improved multi-objective optimization algorithm, which according to the importance of the subject, in addition to speed, also has high accuracy, and by training a neural network after error propagation, it is systematic. We designed that due to the high accuracy and speed of diagnosis, it is a suitable alternative for home diagnosis and similar methods.

Although standalone CNNs have demonstrated a strong performance in melanoma classification, their effectiveness is very sensitive to hyperparameter and architecture choices, which can be time-consuming and expensive to compute, and can easily find a local optimum when searched manually or through grid search. Traditional optimization methods might also fail to cope with non-convex high-dimensional search spaces of deep learning models, premature convergence or overfitting, especially in unbalanced healthcare data like SIIM-ISIC 2020. The metaheuristic algorithms such as Gray Wolf Optimizer (GWO) provide a well-developed alternative to search the various network arrangements to find near-optimal solutions, but the conventional GWO has the disadvantage of its weak exploration capacity and slow convergence in rugged landscapes. These gaps have been filled by the combination of an Improved Gray Wolf Optimizer (IGWO) and CNNs, which also offers improved convergence speed and accuracy of the solution, producing a more dependable and generalizable diagnostic model. Such an automated system can be used clinically as a decision-support tool to dermatologists, particularly in resource-constrained settings or telemedicine, as it can offer fast, repeatable and high-quality screening of melanoma and, in the end, lower diagnostic delays and enhance patient outcomes.

## 2 Dataset description

**Figure 1:**
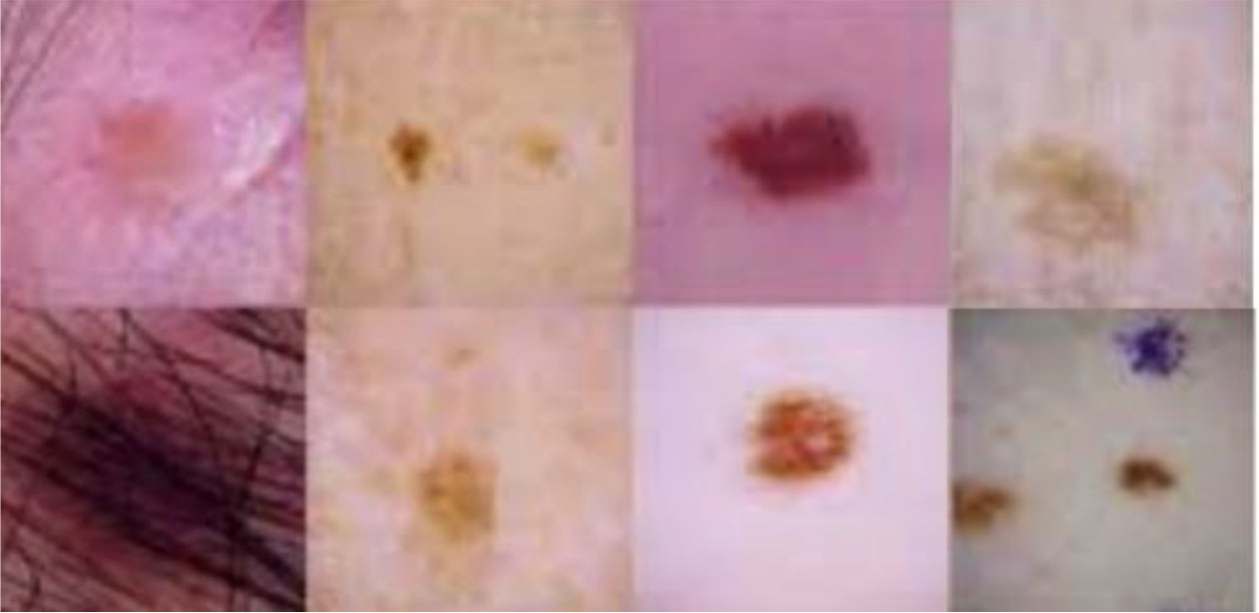
Some samples of the SIIM-ISIC 2020 dataset.

The present study uses SIIM-ISIC 2020 Melanoma Classification for validation. The dataset includes approximately 2,000 patients’ distinct malignant and benign skin lesions represented by 33,126 dermoscopic training photos (21). Each lesion image is linked to an individual patient using a unique patient identifier. Expert opinions, long−term investigations, and histology have been used to validate benign classifications; however, pathology has been employed only to verify cancer diagnoses and treatments. A comprehensive pre-print that hasn’t yet been subjected to peer review is available, detailing all the characteristics of this dataset in great detail. **Figure 1** shows some samples of the SIIM-ISIC 2020 dataset (21).

The Melanoma Institute Australia, the Medical University of Vienna, the University of Athens Medical School, the Memorial Sloan Kettering Cancer Center, the University of Queensland, and Hospital Clinic de Barcelona provided the images and data, which was produced by the International Skin Imaging Collaboration (22). The dataset was organized for the 2020 Summer Kaggle SIIM-ISIC Challenge.

Although the data included in the SIIM-ISIC 2020 Melanoma Classification is a large and heterogeneous dataset of dermoscopic images, it is known to be biased in terms of the number of classes (there are more benign cases than malignant cases) and metadata (notably, disparities in the representation of age, sex, and anatomical location).

To address the issue of class imbalance, we used stratified random splitting in the process of train-validation-test splitting and used class-weighted cross-entropy loss in CNN-training to penalize errors of misclassification of the minority (malignant) group more severely. Moreover, train-test leakage was avoided by making sure that all identifiers of the patient are strictly followed so that no image of a patient would appear in both the training and test sets.

Regarding image−level artifacts (e.g., residual hairs, ruler edges, or uneven lighting), we apply image−based preprocessing with DullRazor hair removal and contrast enhancement, as well as morphological post-processing to minimize irrelevant visual information. Although these steps were applied, the underrepresentation of certain skin types and lesion morphologies constrains the external validity of our model, especially when it is used globally in clinical practice.

Ethically, any autonomous diagnostic system should be regarded solely as a decision−support instrument; the ultimate diagnosis should be supervised by qualified dermatologists to prevent excessive reliance on the results of the algorithm, in particular, in the groups not sufficiently represented in the training sample.

## 3 Improved Gray wolf optimization algorithm

### 3.1 Gray Wolf Optimizer

The gray wolf algorithm simulates leading hierarchies using 4 different wolf kinds, drawing inspiration from the social interactions and hunting behaviors of gray wolves. There is a fairly clear social system among gray wolves. One male and one female, known as alphas, are the group’s leaders. Decisions about hunting, resting, and other activities are primarily the responsibility of the alpha pair. The group makes decisions under the guidance of the alpha.

Interestingly, there is also evidence that the alpha wolf sometimes follows other members of the pack. When a company comes together, everyone can tell who the alpha is because it holds its tail down. The group is expected to abide by the alpha’s instructions. Only alpha wolves within the pack are permitted to select a mate.

It’s fascinating to consider that the alpha is not always the company’s strongest member, but rather the one who manages the group the best. This demonstrates that a group’s structure and functioning are far more significant than its level of strength. Beta is the next position in the hierarchy of gray wolves. The alpha’s adviser and group coordinator is called the beta.

While reporting back to Alpha, Beta carries out Alpha’s instructions across the organization. Omega is the lowest ranked gray wolf. The victimized character is Omega. The last pack of wolves to be given permission to eat is them. An unalpha, beta, or omega wolf is referred to as subordinate (or delta in some sources).

Although it rules over the Omega, the Delta Wolf should answer to the Alpha and Beta. In addition to the social hierarchy, there are three phases of gray−wolf hunting: tracking, pursuing, and reaching the prey. We rank alpha as the perfect approach, beta as the second-best response, and delta as the third-best response when modeling the social structure of wolves. We classify the remaining potential answer as Omega. The fourth group comes after the first three groups since alpha, beta, and delta are what drive optimization. The following connections are used to mimic how wolves behave during sieges:

(1) $$\vec{D}=|\vec{C}\times \vec{X_{p}}(t)-\vec{X}(t)|$$

Equation 2 shows the updating process formulation:

(2) $$\vec{X}(t+1)=\vec{X}(t)-\vec{A}\times \vec{D}$$

In these relationships, *t* is the present iteration number, *A* and *C* denote the coefficient, $\vec{X_{p}}(t)$ is the prey situation vector, and *X* describes the situation vector of a wolf.

To calculate vectors *A* and *C*, Equations 3 and 4 are used, respectively.

(3) $$\vec{A}=2\vec{a}\times \vec{r}-\vec{a}$$

(4) $$\vec{C}=2\times \vec{r}$$

The vector *a* reduces between 0 to 2 linearly throughout the generation period in both exploration and exploitation stages. *r* represents a random vector from 0 to 1. Due to the randomness of vectors $r_{1}$ and $r_{2}$, wolves must alter their situations in the space containing the food randomly based on Equations 5–11:

(5) $${\vec{D}}_{\alpha }=|{\vec{C}}_{1}\times {\vec{X}}_{\alpha }(t)-\vec{X}|$$

(6) $${\vec{D}}_{\beta }=|{\vec{C}}_{1}\times {\vec{X}}_{\beta }(t)-\vec{X}|$$

(7) $${\vec{D}}_{\delta }=|{\vec{C}}_{1}\times {\vec{X}}_{\delta }(t)-\vec{X}|$$

(8) $${\vec{X}}_{1}(t)={\vec{X}}_{\alpha }(t)-{\vec{A}}_{1}\times {\vec{D}}_{\alpha }$$

(9) $${\vec{X}}_{2}(t)={\vec{X}}_{\beta }(t)-{\vec{A}}_{1}\times {\vec{D}}_{\beta }$$

(10) $${\vec{X}}_{3}(t)={\vec{X}}_{\delta }(t)-{\vec{A}}_{1}\times {\vec{D}}_{\delta }$$

(11) $$\vec{X}(t+1)=\frac{{\vec{X}}_{1}+{\vec{X}}_{2}+{\vec{X}}_{3}}{3}$$

The same idea may be used to a search space with n dimensions. In higher−dimensional search spaces, gray wolves move toward the optimal solution relative to the dimensional boundaries. Alphas frequently take the lead in gray wolf hunts.

Sometimes, beta and delta wolves will also join the hunt. We keep three of the optimal answer found to replicate this pattern, and we compel additional search agents to modify their positions in accordance with the positions of the top search agents by using Equation 7.

When the prey is halted in this method, the exploitation or assault stage is implemented by changing the amount of the parameter from 2 to 1. Additionally, depending on ∝, the amount of A diminishes.

**Figure 2:**
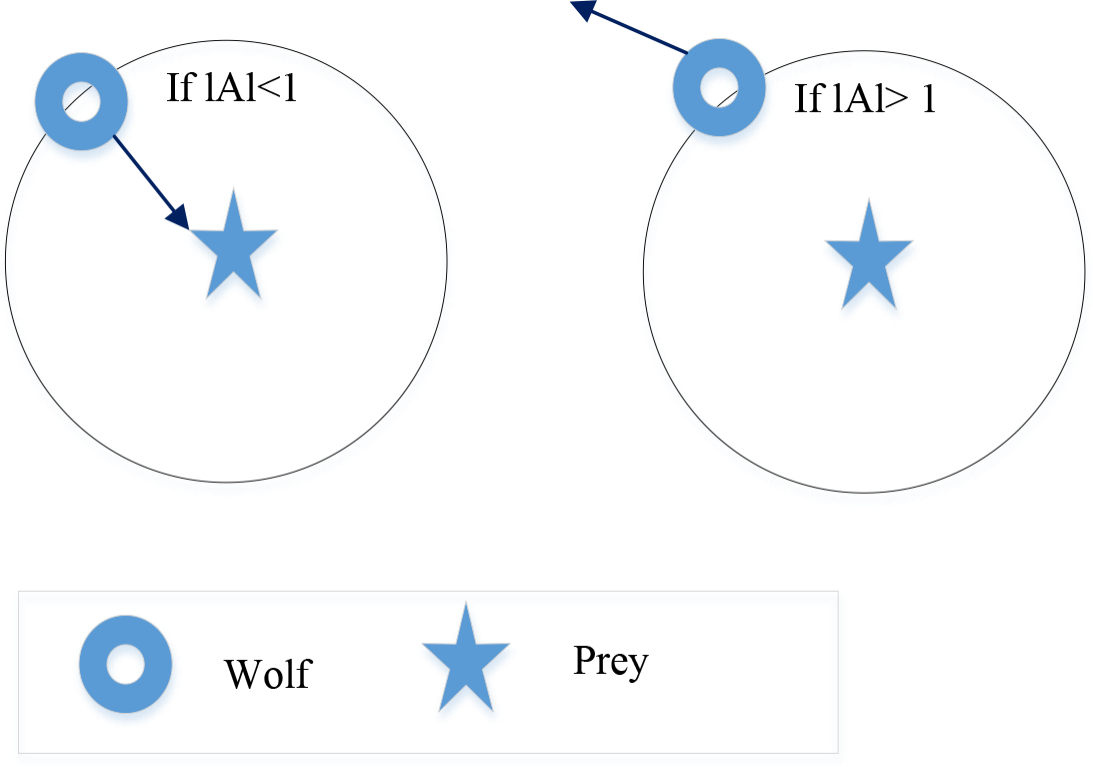
Exploration phase versus exploitation.

Wolves are compelled to attack their victim by lowering the value of *A*. Additionally, an identifying step is included to prevent becoming trapped in a local minimum. When looking for prey, wolves maintain a distance from one another. However, when attacking and working together, they become close to one another. We employ a vector *A* with random values greater than 1 or less than -1 to model this divergence. **Figure 2** shows the exploration and exploitation.

**Figure 3:**
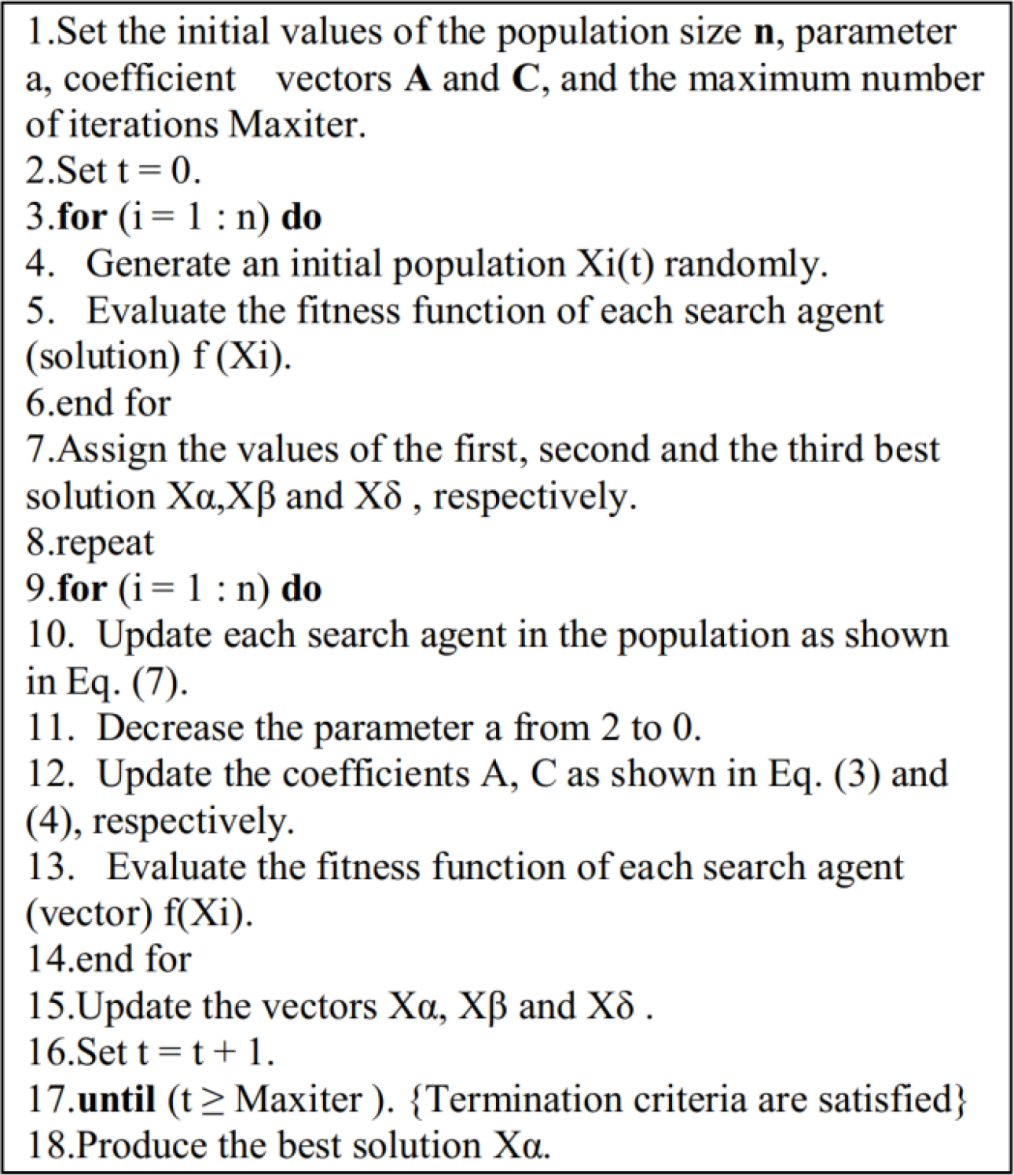
Gray Wolf Algorithm Pseudocode.

The amount of *C* also plays a significant role in the identifying procedure. A random amount in the range [0,2] serves as the amount of this vector. This random value *C* influences the calculation of distance, intensifying effects when C>1 and weakening them when C<1. This vector may also be seen as the result of natural barriers that hinder a predator from accessing its victim. Individual pseudocode of the Gray Wolf Algorithm is represented in **Figure 3**.

### 3.2 Improved Gray wolf optimization

The population in the gray wolf algorithm tends toward alpha, beta, and delta wolves, or ideal solutions. The best particle is known as alpha, and in every generation, the best particles are chosen in accordance with the objective function. Every dimension of the innovative situation is equivalent to the mean of the corresponding dimensions of the best components, as discussed earlier in Equations 1-7. The passage of particles through each step is non-greedy and is carried out independently of objective level.

The objective of this method is to modify the initial population to achieve higher objective values. In each cycle, omega wolves are located and eliminated from the starting population. We replace the omega wolves with the optimal particles or with other wolves randomly chosen from the population to create a population with high objective in order to converge to the solution more quickly. Any objective level could be found in random particles. The wolves are put randomly in the first generation with a greater likelihood, and with a greater likelihood in the final generation based on objective.

Additionally, the objective value at the new position is compared with that of the previous best position with the highest objective in each generation to shift the placement of the particles. The position will alter if the new place is more suitable; else, the most suitable situation will be chosen as the new situation.

In order to achieve the maximum level of transparency and reproducibility of the proposed Improved Gray Wolf Optimizer (IGWO), we explicitly formulate its mechanics of operation in the CNN hyperparameter optimization environment. Each candidate solution explores a search space defined by four parameters: the number of filters in the three convolutional layers (f1, f2, f3), which is an integer and an operation in [16, 256], and the dropout rate (d) in the fully connected layer, which is a continuous variable in [0.2, 0.8]. The objective minimized by IGWO is the validation loss of the CNN with the following hyperparameters, which is trained 50 epochs on a fixed stratified subset (15%) of training data.

**Figure 4:**
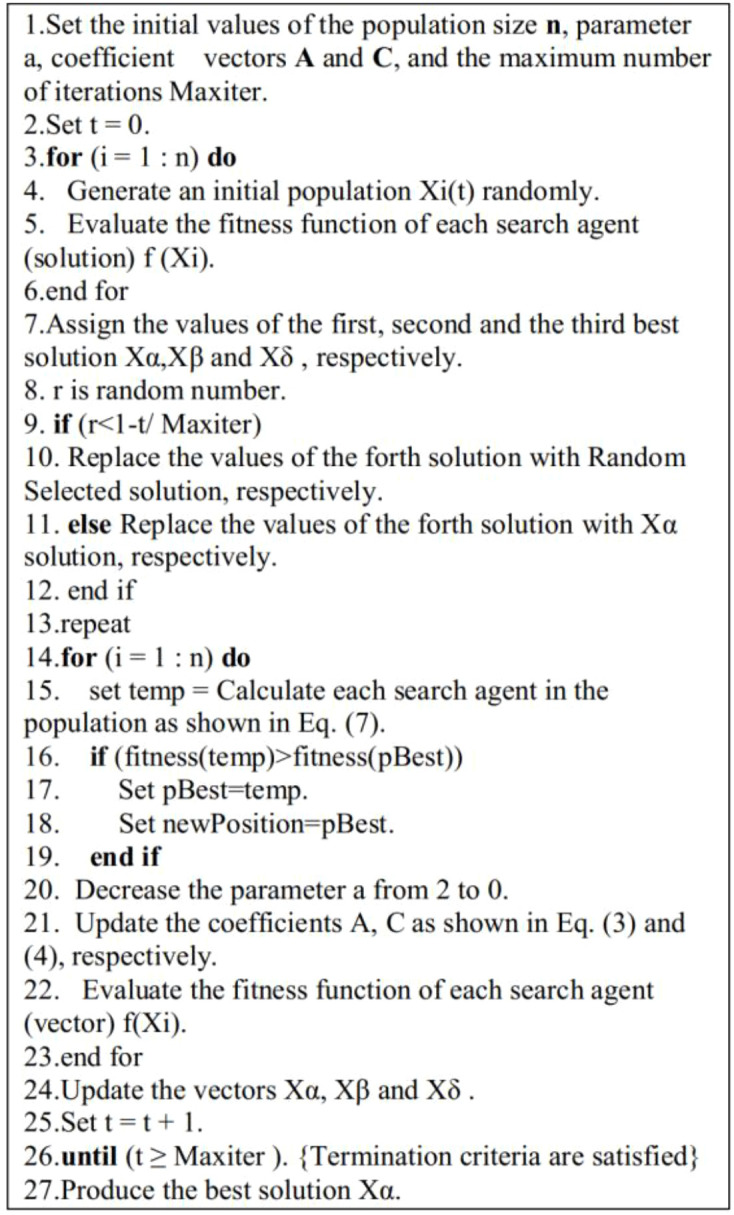
Pseudocode of the proposed algorithm.

IGWO algorithm has 30 search agents as its population and 200 iterations as its maximum. At every step of iteration, the agents are ranked in terms of their fitness and the lowest 30% (r = 0.3) becomes omega wolves and is then removed. They are regenerated over time by a probabilistic process, i.e. with a 70 percent chance that any eliminated agent is replaced by an elite solution (weighted by its fitness) in the alpha, beta, or delta wolves, and with 30 percent probability that any agent that is killed is randomly replaced by a stochastically resampled candidate generated by introducing Gaussian noise to the position of the alpha wolf, where the noise magnitude decreases as the iteration number increases, to trade off exploration with exploitation. This stepwise description explains the dynamics of search of the algorithm such that it can be replicated and benchmarked by other scientists. The pseudocode of the suggested algorithm is presented in **Figure 4**.

To formalize the novelty and ensure the reproducibility of the Improved Gray Wolf Optimizer (IGWO), we characterize the major adaptations to the standard GWO. In standard GWO, every search agent (including poorly performing omega wolves) contributes to the position−update process via Equations 7-11, which can slow down convergence and encourage stagnation in high-dimensional or rugged search spaces.

In IGWO, we present a fitness-based omega regeneration mechanism which is dynamically used to replace poor performing omega agents. Suppose the population at iteration *t* is $X(t)=\{x_{1},x_{2},\ldots ,x_{N}\}$ and the fitness is $F=\{f_{1},f_{2},\ldots ,f_{N}\}$. Once the top three solutions $x_{\alpha },x_{\beta },x_{\delta }$ have been found, the rest of the agents (omegas) are sorted by fitness. Bottom *r*. Nagents (with r∈(0,1], e.g., r=0.3) are eliminated.

Each eliminated agent $x_{i}$ is recombined as:


$$x_{i}^{\text{new}}=\{\begin{array}{cc} x_{j}, & \text{with probability }p=0.7\\ x_{\text{rand}}, & \text{with probability }1-p \end{array}$$

, in which $x_{j}\in \{x_{\alpha },x_{\beta },x_{\delta }\}$ in the set of $x_{\alpha },x_{\beta },x_{\delta }$ is randomly chosen with a probability that is proportional to its rank in the fitness table, and $x_{\text{rand}}$ is a new candidate randomly chosen in the search space, with a Gaussian perturbation: $x_{\text{rand}}=x_{\alpha }+\sigma \cdot N(0,I),\sigma =\frac{1}{t+1}$. This strategy enhances exploitation without compromising exploration, especially in later iterations.

## 4 Image pre-processing

### 4.1 Remove hair, bubbles and oil from the image

Skin images typically exhibit Gaussian noise, independent of the image content; this noise is reduced using a Gaussian low-pass filter. Subsequently, artifacts such as hair and, if present, bubbles (e.g., in water or oil) are removed from the image. Hair and similar artifacts can be effectively removed using the DullRazor methodology. During hair removal, dark lines (representing hairs) in the skin image are identified and replaced using a statistical averaging technique. The zoning process separates the irrelevant background from the region of interest. The pseudocode of Dullrazor methodology is given below (23). **Algorithm 1** illustrates the pseudocode of Dullrazor.

Algorithm 1 **Pseudocode of Dullrazor.**

Stage 1: Expand the image, then erode it to remove the fine features.Stage 2: Determine how the resulting image differs from the primary image.Stage 3: Erode and dilate the mask of difference to get rid of the noise.Stage 4: Create a boolean mask that includes the locations of the artifacts in stage four.Stage 5: From the primary image, replace the cells masked by mask with those that correspond to it.

### 4.2 Contrast enhancement

After segmenting the lesion from surrounding tissue and removing noise from relevant features, we proceed to the preprocessing stage aimed at improving image quality. Many medical images suffer from low brightness and overall quality. Therefore, enhancing image quality before further processing is essential. Histogram expansion transfer functions, applied generally to the entire image, are one method used for this purpose.

Dermoscopy images often exhibit low contrast, hindering subsequent processing. This issue arises from several factors, including low-quality imaging devices and cameras, suboptimal operator technique during photography, environmental conditions, and image noise. In such cases, crucial image features can become obscured, significantly complicating processing (24). Contrast enhancement offers a solution to these issues with contrast consistency.

**Figure 5:**
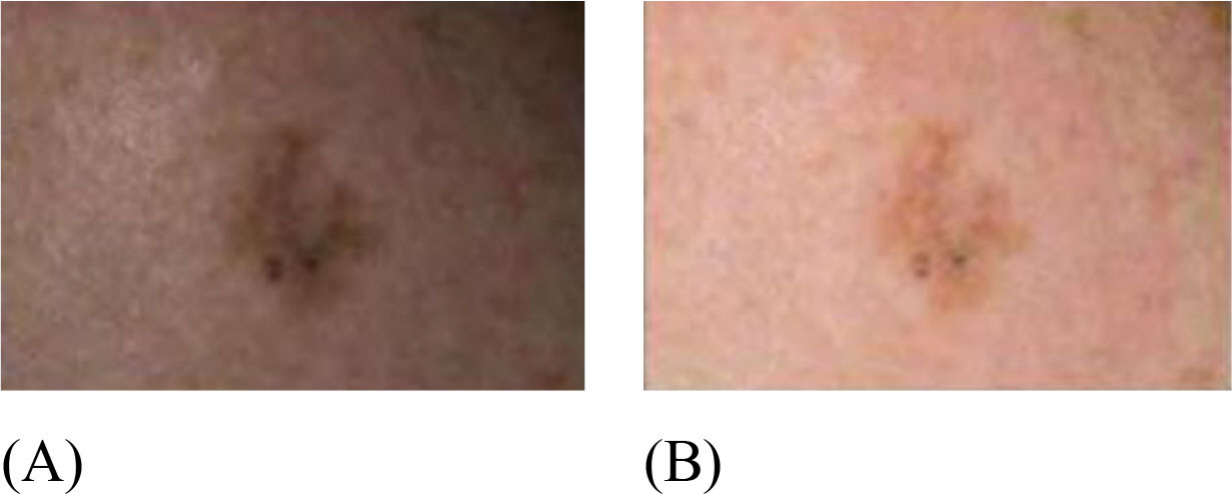
Sample of contrast enhancement for an input image: **(A)** raw image, and **(B)** enhanced image.

In this study, image contrast enhancement is employed to improve the visibility and detail of cancerous lesion locations. This research applies a global enhancement technique using a Lookup Table (LUT). An 8-bit lookup table is utilized for image classification and saving the processed images to disk. The strategy is typically implemented as Equation 12:

(12) $$pdf_{o}=\frac{pdf_{i}-\underline{pdf}}{\bar{pdf}-\underline{pdf}}$$

where, $pdf_{i}$ and $pdf_{o}$ describe, in turn, the probability density function for input raw image and output enhanced image, and $\underline{pdf}$ and $\bar{pdf}$ signify, in turn, the smallest and the most likelihood density level. **Figure 5** provides an example of contrast enhancement applied to an input image.

## 5 Segmentation

### 5.1 Segmentation based on entropy

In computer vision, the technique of partitioning a digital image (a collection of pixels, also referred to as ‘image objects’) into distinct segments or regions is known as image segmentation. Image feature extraction aims to facilitate imaging techniques by simplifying or transforming an image’s initial representation into a more meaningful format.

Image segmentation is typically used to locate objects and identify boundaries (lines and curves) within an image. More precisely, image segmentation is the procedure of assigning a label to all pixels in a digital image such that pixels with the same label share common characteristics.

The final outcome of image processing in this context is a set of segmented parts or regions. Combining all these regions reconstructs the original image. Additionally, contours derived from the digital image can be an outcome of the image segmentation procedure.

Pixels within a particular region or segment (obtained from image segmentation) are comparable to one another based on various determined attributes like Color, Intensity, or Texture. Conversely, adjacent regions or sections will differ significantly from this specific part due to variations in the computed features or attributes. Image thresholding is one of the most commonly used techniques for image segmentation. The threshold value in this approach is specified using the following formula (Equation 13).

(13) $$TH=[th_{1},\;th_{2},\;\ldots ,\;th_{m-1}]$$

In this condition, the Kapur entropy technique can be formulated as Equation 14:

(14) $$\begin{array}{l} J_{max}(TH)=\sum_{i=1}^{m}H_{i}^{c}\\ c=\{\begin{array}{cc} 1,2,3, & \begin{array}{ccc} if & RGB\;scale & image \end{array}\\ 1, & \begin{array}{cccc} if & Gray & scale & image \end{array} \end{array} \end{array}$$

where, $H_{i}^{c}$ defines the image’s $i^{th}$ entropy and is achieved based on Equation 15:

(15) $$\begin{array}{c} H_{1}^{c}=\sum_{i=1}^{th}\;\frac{ph_{i}^{c}}{\omega_{0}^{c}}\ln(\frac{ph_{i}^{c}}{\omega_{0}^{c}}),\\ \vdots \\ H_{m}^{c}=\sum_{i=th+m}^{L}\;\frac{ph_{i}^{c}}{\omega_{m-1}^{c}}\ln(\frac{ph_{i}^{c}}{\omega_{m-1}^{c}}), \end{array}$$

where, $ph_{i}^{c}$ describes the image probability density function, $\omega_{i}^{c}$ describes the $i^{th}$ probability density function for $c_{i}$, and ln(.) presents the natural logarithm.

This study tunes threshold values until the ideal threshold is attained using a novel particle thermal exchange optimization method.

### 5.2 Thresholding by means of Improved Gray wolf optimization algorithm

In the suggested method, each candidate represents a choice for the threshold value and image segmentation. The population is defined in Equation 16:

(16) $$\begin{array}{l} X={\left[x_{1}^{c},x_{2}^{c},\ldots ,x_{candidate_{m}}^{c}\right]}^{T},\\ x_{i}^{c}=[th_{1}^{c},th_{2}^{c},\ldots ,th_{m}^{c}],\\ c=1,2,3 \end{array}$$

The population size is described by *X*, the i-th element of *X* is denoted by $x_{i}$, the transposition sign is denoted by *T*, and the set *c* is specified for RGB pictures though c=1 is chosen for images for grayscale color space. In the current investigation, the search space bounds refer to the image intensity levels, ranging from 0 to 255.

*TH* defines the size of the search space. The algorithm is allowed a maximum of 200 iterations. The algorithm will end after the objective function, ( $J_{max}$) has been maximized. To ensure a fair analysis, the procedure is repeated 25 times, and the mean value is considered the optimal threshold. In the current study, various online datasets are examined to evaluate the performance of the recommended segmentation process.

Mathematical morphology, based on the Kapur approach, is used to determine the overall procedure, identify regions, or reveal image limitations after thresholding. The indication of image shape, often determined by its form, is checked (25).

Morphological operations simplify image features by preserving shape criteria while removing superfluous information. Morphological operations are a relatively new method based on Makowski’s principles of sets and algebraic processes. This method is particularly useful in ecological studies and ecosystem research. This article uses three morphological operations—opening, closure, and filling—to remove additional sections related to skin cancer.

To achieve this, empty holes in the threshold image are first filled using the filling operator. This is achieved based on Equation 17 as follows:

(17) $$X_{k}=(X_{k-1}⊕e)\cap A^{c},k=1,2,3\ldots$$

where, *A* and *e* signify the regions and the structuring parameter.

In this study, *e* is a 3×3 matrix.

The second operator, mathematical opening, is used to eliminate small details without altering the remaining information. This can be achieved based on Equation 18:

(18) A⚬e=(A⊖e)⊕e

The final operation is based on mathematical closing. This process is employed to connect narrow gaps or regions. Mathematical closing is formulated by Equation 19:

(19) A•e=(A⊕e)⊖e

**Figure 6:**
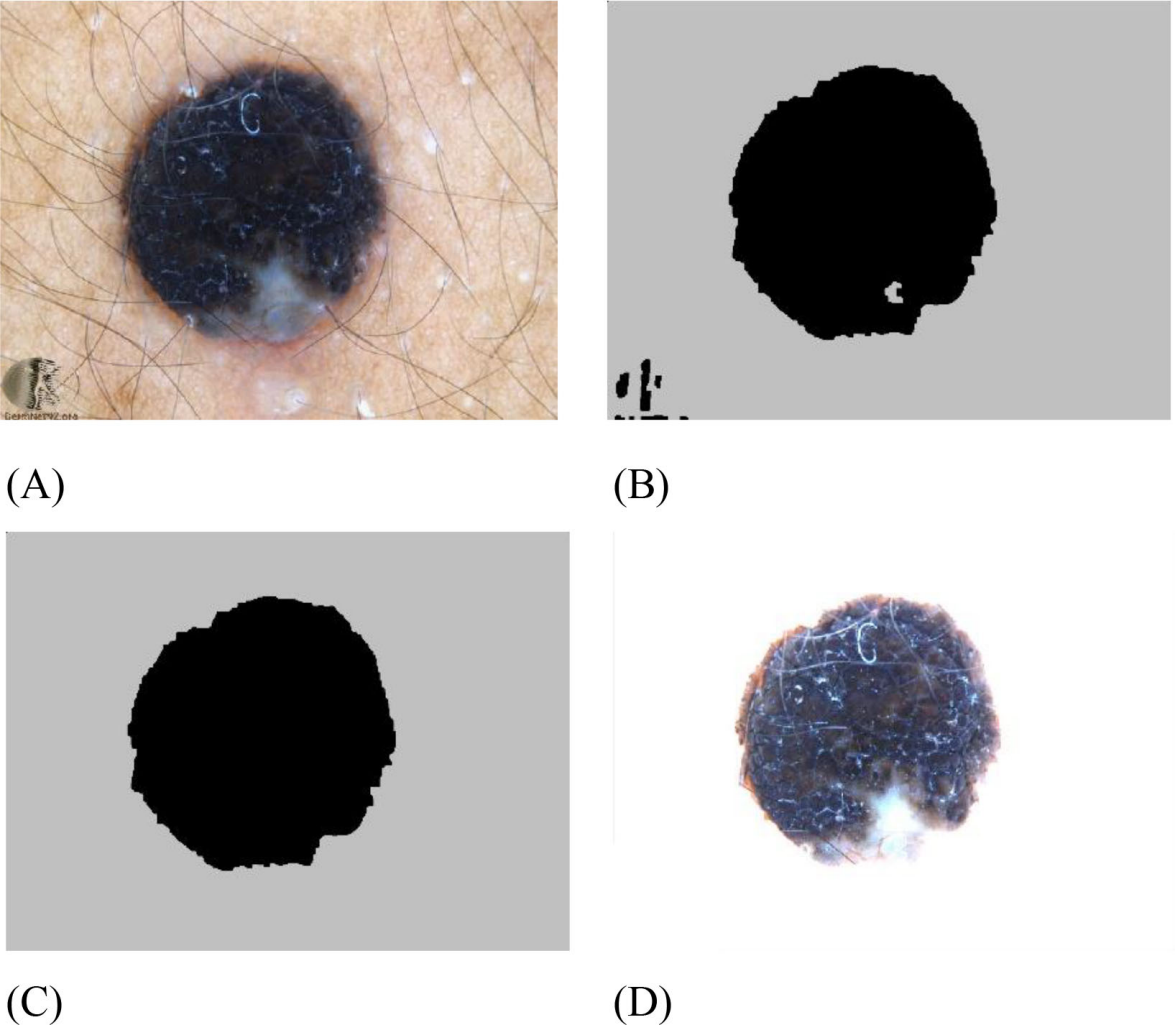
Example result for skin image dermoscopy classification: **(A)** raw image, **(B)** image after Kapur segmentation, **(C)** image **(B)** after calculated morphology, **(D)** segmented image.

**Figure 6** shows an example effect of skin image dermoscopy segmentation.

**Figure 7:**
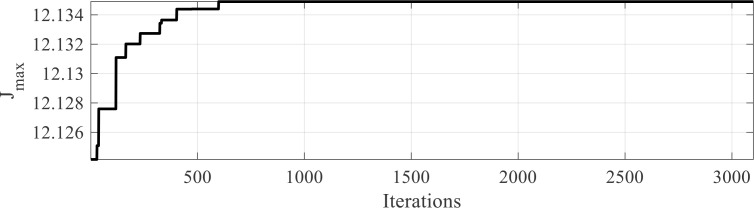
Results of entropy maximizing for thresholding of the image.

As observed from the results, the sample output demonstrates good performance for the image. **Figure 7** demonstrates the effects of entropy maximization in image thresholding.

It can be observed that the proposed method achieved maximum entropy after approximately 607 iterations.

## 6 Convolutional neural network

For the research’s architectural design and analysis, images of a consistent size are required. Consequently, all images were rescaled to 225×225 pixels before being used to train the CNN. During network training, the error between the network’s actual and predicted outputs is minimized. This is accomplished by adjusting the network’s weights and biases, which are its learnable parameters. In this work, a supervised approach was used for training. In this approach, a supervisor guides the training process, teaching the network to learn correctly. In other words, the network is presented with sample input-output pairs.

The network’s output is then compared to the target output to determine the magnitude of the error. Subsequently, the biases and weights are adjusted to minimize this error value. Network parameters can be trained in one of two ways: sequentially (stochastically), updating after each training sample, or batch-wise, updating after processing all training data.

**Figure 8:**
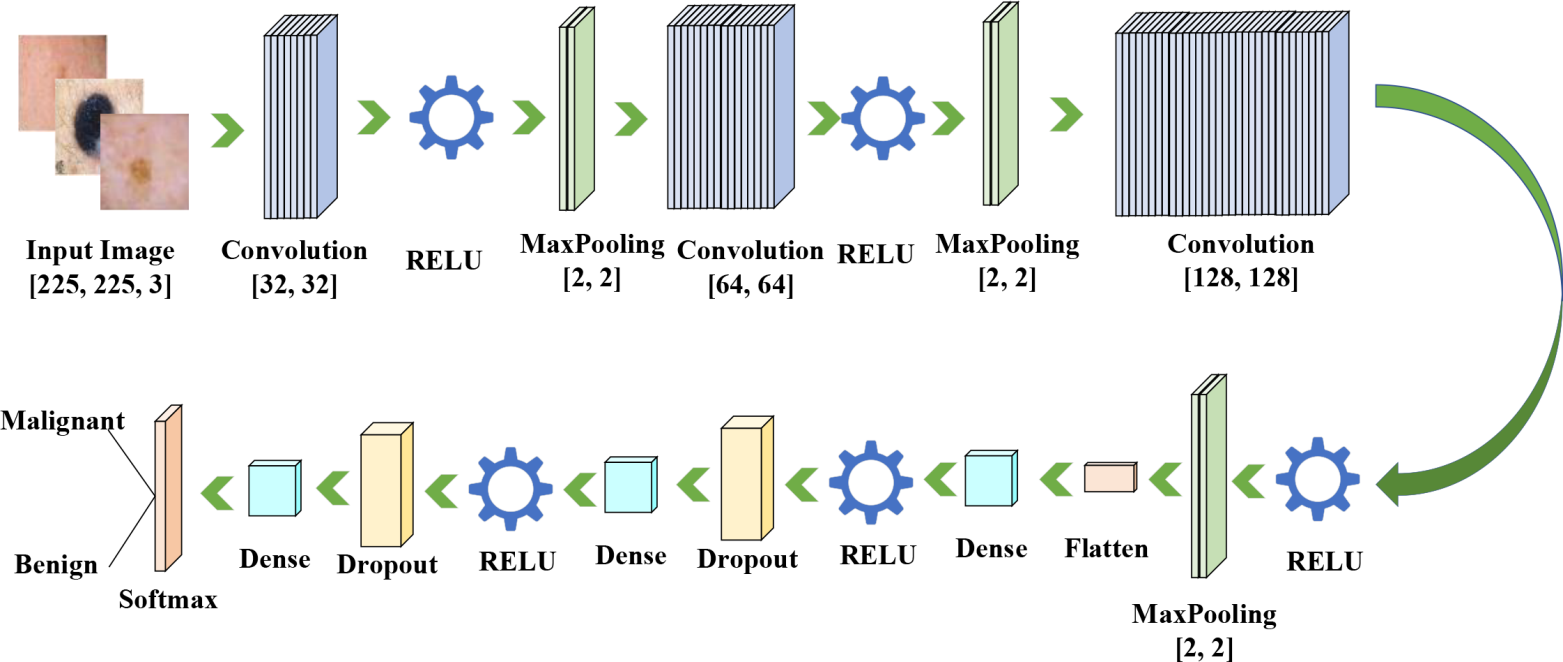
Full architecture of the suggested CNN model.

The sequential approach uses less memory but is less stable, as each training sample can influence network parameters differently. The batch-wise approach is more stable, though it requires more memory to store the updates. Consequently, the batch mode of training is employed. In this work, the dataset images were trained using batches of size 32. The deep neural network architecture consists of three convolutional layers and three max-pooling layers. **Figure 8** illustrates the overall architecture.

The convolutional layer contains the primary kernel of the CNN, and its output can be viewed as a 3-dimensional matrix of neurons. Convolution is a signal processing method used to apply convolutional operations to the inputs received from neurons. The filter size of the convolutional layers is a crucial variable. In the suggested model, three layers with filter sizes of 32×32, 64×64, and 128×128 are employed. A pooling layer is used after the convolutional layer to reduce the spatial dimensions (depth).

This increases network speed and reduces the number of parameters. Pooling layers reduce the number of feature maps (output layers from filters). For this purpose, a 2×2 filter is employed in this investigation. The primary concept is to subsample the input image to decrease computational cost, storage overhead, and the number of network variables.

The neural network’s sensitivity is also reduced by this downsampling of the input image. Similar to convolutional layers, neurons in a pooling layer are connected to specific output neurons from the preceding layer. Pooling layers are employed for sampling to reduce dimensionality and increase computation speed. In this investigation, max-pooling layers with a window size of 2×2 are used. The largest pixel value within the 2×2 window is selected and passed to the next layer.

**Figure 9:**
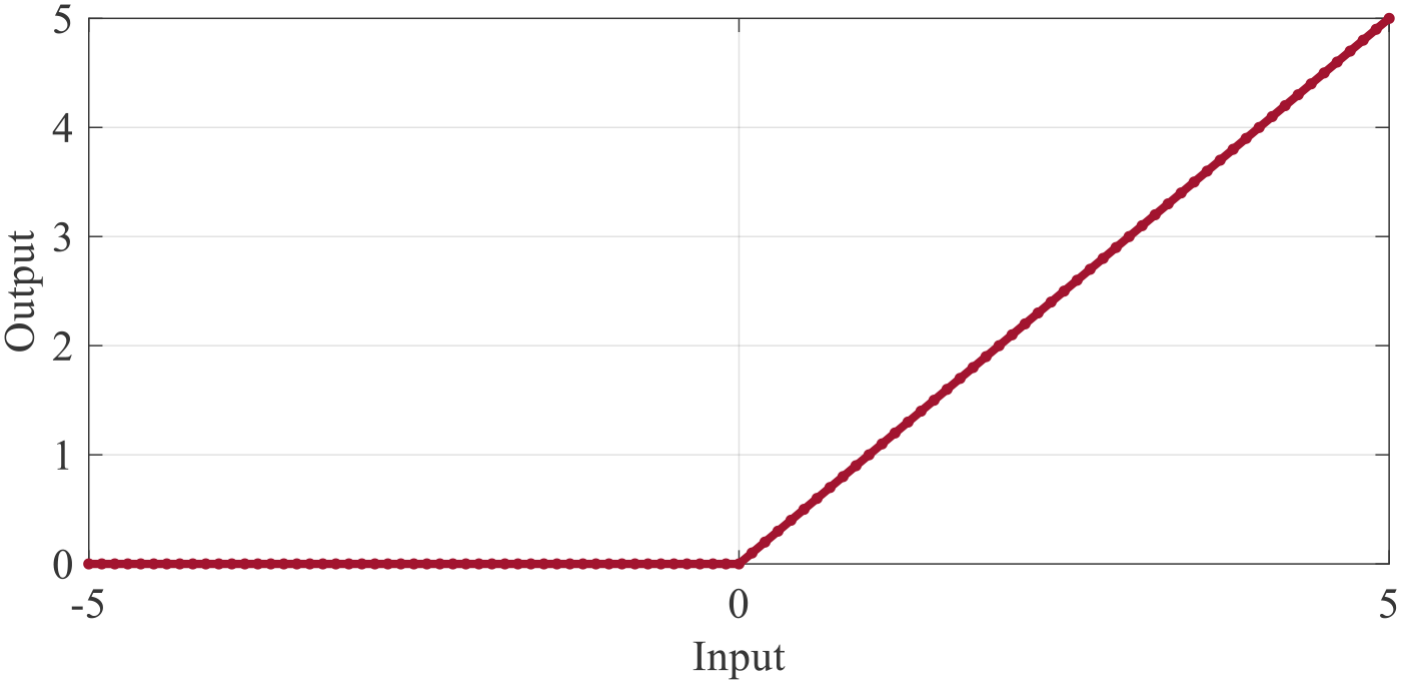
Profile of the rectified linear unit.

An activation layer is utilized after the convolutional layers to introduce non-linearities, complementing the linear operations performed in the preceding mathematical layers. For this purpose, this study employs Rectified Linear Unit (ReLU) layers. This is due to the efficiency of ReLU’s computation, enabling the network to learn more quickly without compromising accuracy. This effectiveness profile of the ReLU activation function is illustrated in **Figure 9**.

All of the negative activations in the input pictures are turned into zeros by the RELU layer, which applies the function, f(x), to all of them. The use of this layer enhances the network’s and model’s non-linear properties without altering the convolutional layers themselves.

The ‘Dropout’ layer was incorporated during training; based on a specific probability, the output of some neurons was randomly set to zero. This procedure creates distinct subnetworks, forcing each to learn robust features independently, thus avoiding reliance on specific neurons. The dropout approach thus helps to avoid the overfitting issue. Fully connected deep networks often have a large number of parameters, leading to long training times and an increased risk of premature convergence. Therefore, dropout layers are utilized among the fully-connected layers to reduce the number of parameters and mitigate overfitting.

After the convolutional and pooling layers, a large amount of feature data, typically with reduced spatial dimensions, is generated. Since the input image labels are known when using a Softmax classifier, the network can be trained for image classification by feeding it all trained images and their corresponding labels. During training, the system searches for the optimal values of uncertain variables, specifically filter weights and layer coefficients, to achieve the lowest possible classification error.

Weight optimization considers the ‘Flatten’ layer, which transforms multi-dimensional convolutional layer tensors into one-dimensional tensors, and utilizes the RMSprop Optimizer, employed for evaluating various activation functions in TensorFlow. The network is tested using the images from the dataset that were not used during training. In this testing scenario, the output of the layers serves as the extracted feature vector for the image.

To interpret certain data portions, the CNN compares the image’s feature vector with a characteristics matrix at the final stage. However, to classify the data, a set of probabilities is required. Softmax is a common function used for normalizing probabilities across a conventional range (0 to 1).

As noted, the RMSprop Optimizer’ approach is employed to determine the weights optimally. The system’s optimization process focuses on minimizing cross-entropy, which can be formulated by Equation 20:

(20) $$L=\sum_{j=1}^{N}\sum_{i=1}^{M}-f_{j}^{i}logy_{j}^{i}$$

where, *N* signifies the quantity of the examples, $f_{j}=(0,\ldots ,0,\underset{k}{\underset{︸}{1,\ldots ,1}},0,\ldots ,0)$ defines the favorite vector, and $\;y_{j}$ represents the gained yielded vector of the class number *m* and has been reached as Equation 21:

(21) $$y_{j}^{\left(i\right)}=\frac{e^{g_{j}}}{\sum_{i=1}^{M}e^{g_{i}}}$$

With assuming a weight penalty ( $w_{p}$), the system's optimization process is turned into Equation 22:

(22) $$L=\sum_{j=1}^{N}\sum_{i=1}^{M}-f_{j}^{i}logy_{j}^{i}+\frac{w_{p}}{2}\sum_{K}\sum_{O}\omega_{k,l}^{2}$$

In this equation, $\omega_{k}$specifies the weight of a link, *O* denotes the overall number of layers, and *K* characterizes the connections inside a layer, *l*.

Several research papers offer insights into the optimal CNN structure. For this reason, the proposed Improved Gray Wolf Optimizer (IGWO) is developed as a novel metaheuristic-based optimization approach.

To prevent system faults in this article, we first define the minimum (min) and maximum (max) bounds of the method. Here, min is set to 1 (the minimum allowed value for max-pooling window size), while max specifies the sliding window size.

The input data’s spatial extent may exceed the sliding window size. The CNN’s hyperparameter variables are represented by ten real numbers. These solutions are then evaluated. In this work, the cost function used for the optimized convolutional neural network was based on accuracy. It should be highlighted that the entire process incurs significant computational cost. This is because the entire population must be trained using the backpropagation technique on the (melanoma) dataset. In this study, Equation 23 and Equation 24 are chosen as optimization parameters where the components can be defined by Equation 25 and Equation 26:

(23) $$W=[w_{1},w_{2},\ldots ,w_{p}]$$

(24) $$A=[a_{1},a_{2},\ldots ,a_{A}]$$

(25) $$w_{n}=[w_{1n},w_{2n},\ldots ,w_{Ln}]$$

$b_{n}=[b_{1n},b_{2n},\ldots ,b_{Ln}]$

l=1,2,…,L

(26) n=1,2,…,A

Here, *n* specifies the individual quantity, the term *l* stands for the layer index, *A* represents the overall amount of layers and agents, and, accordingly, $w_{in}$ defines the definition of the weight in a layer *i*. The CNN’s error cost function is defined in Equation 27.

(27) $$E=\frac{1}{T}\sum_{i=1}^{T}\sum_{j=1}^{k}{\left(f_{ji}-y_{ji}\right)}^{2}$$

where, *T* denotes the quantity of training data, *k* specifies the number of output layers, and $f_{ji}$ and $y_{ji}$ represent, in turn, the intended and output value of the CNN. This approach can easily become trapped in local minima, a problem that metaheuristics aim to resolve. Metaheuristics also do not require a backpropagation phase, which incurs a significant computational cost.

In this research, the proposed improved gray wolf optimizer has been employed here for minimizing the term *E* in Eq. (27).

## 7 Results and discussions

### 7.1 System configuration

The proposed methodology was implemented on the MATLAB R2017b platform, using hardware that included 16GB RAM and an NVIDIA GeForce RTX 3060 GPU. The SIIM-ISIC 2020 dataset is employed to evaluate the suggested diagnostic system for melanoma classification. The parameters for the recommended CNN/IGWO approach were determined through trial and error to maximize efficiency.

### 7.2 Measurement indicators

Accuracy, F1 score, precision, Area Under the ROC Curve (AUC), and specificity were employed as performance criteria to compare and assess approaches for the SIIM-ISIC 2020 dataset’s melanoma classification. These performance metrics are derived from the primary components: True Positives (TP), True Negatives (TN), False Positives (FP), and False Negatives (FN).

The proposed CNN includes three convolutional blocks, each consisting of a Conv2D layer, ReLU activation, and a 2x2 MaxPooling block.

The filter settings are as follows:

Block 1: 32 3x3 filters, input size (225, 225, 3).

Block 2: 64 filters of size 3x3.

Block 3: 128 filters of size 3x3.

Following the convolutional blocks, the feature maps are flattened to a 1D format and then passed through two fully connected (dense) layers:

Dense Layer 1: 128 neurons having ReLU activation.

Training density 2 (output): 2-neurons binary (benign vs. malignant) Softmax-activated neurons.

To mitigate overfitting, an intermediate Dropout layer (rate = 0.5) is inserted between the two dense layers.

The model uses categorical cross-entropy as its loss function, with class weights applied to address the imbalance in the SIIM-ISIC 2020 dataset, where benign cases significantly outnumber malignant ones. Class weights are determined to be inversely related to the frequency of each class in the training set.

RMSprop (learning rate = 0.001, ρ = 0.9, ϵ = 1e−7), backprop optimizer was used in the evaluation in the IGWO and the training was conducted in 32 backprop batches of 50 epochs of each IGWO candidate solution. The evaluation of each candidate employed early stopping with a patience of 10 epochs to prevent overfitting. Notably, IGWO optimizes only two hyperparameters:

- Multiplicity of 1D-filter in every convolutional layer (search: [16, 256]).
- Per cent drop out of completely connected component (range of search [0.2, 0.8]).

All other architectural parameters (number of layers, kernel size, activation functions, and pooling strategy) are fixed and not optimized. This performance improvement is attributed to IGWO’s capability in locating excellent hyperparameter settings, rather than an inherently more advantageous architecture. The final model has a total of 3,527,426 trainable parameters. This information facilitates independent replication and validation, confirming that the advertised 98.47% test accuracy results from a combination of the CNN backbone and IGWO’s search performance.

The hyperparameters of the proposed CNN/IGWO framework were determined in a two-step process: (1) setting non-optimizing training parameters and (2) optimizing key structural hyperparameters using IGWO. Specifically, the RMSprop optimizer learning rate was initialized to 0.001 because large-scale preliminary experiments in the range $\left[10^{-5},10^{-2}\right]$ demonstrate that this value lies within the ballpoint range.

We discovered that loss convergence was erratic at learning rate 1.01 and 1.02 and slow training and irrelevant minima at learning rate $10^{-4}$. Significant oscillations in the convergence rate were consistently observed at 0.001, a finding supported by general practice in dermoscopic image classification. Other training variables like the batch size (32), number of epochs (50), RMSprop decay (ρ = 0.9) and the numerical stability constant ( ϵ = 1e−7) were also pinned down by this grid search and kept constant between IGWO candidate evaluations so that an equal comparison could be made between them. However, IGWO dynamically optimized two important CNN hyperparameters:

Filter sizes for the three convolutional layers (search space: [16, 256] per layer, integer values),

Fully connected Dropout rate (search space: [0.2,0.8], continuous).

IGWO was particularly concerned with each candidate solution, which has 4-dimensional input $\left[f_{1},f_{2},f_{3},d\right]$, each $f_{i}$ specifies filters at the ith layer and d specifies the dropout rate. All candidates were trained for 50 epochs using a CNN with a constant training protocol. Validation accuracy was then evaluated on a 15% stratified hold-out set, separate from the test data. The IGWO algorithm then optimized this objective function using a population size of 30.

Therefore, several structural choices (e.g., number of layers, kernel size = 3×3, activation functions like ReLU or Softmax) were made *a priori* based on dermatological imaging literature to simplify model interpretation, rather than being hand-tuned. Crucial hyperparameters influencing performance, however, were chosen by IGWO over 25 independent runs. The reported configuration achieving 98.47% test accuracy is the best performing one:

- Filters: [32, 64, 128].
- Dropout: 0.54.

The precision of the offered CNN/IGWO approach, analogous to its use in document retrieval, can be understood as the proportion of relevant records it returns as defined in Equation 28.

(28) $$Precision=\frac{TP}{TP+FP}$$

Sensitivity is calculated based on the proportion of actual positive findings correctly identified by the model. It is defined as the proportion of correct predictions relative to all predictions as defined in Equation 29.

(29) $$Sensitivity=\frac{TP}{TP+FN}$$

The F1-score represents the harmonic mean of precision and sensitivity. The F1-score is calculated as the weighted harmonicmean of precision and sensitivity as Equation 30.

(30) $$F1-score=\frac{\left(2\times Precision\times Sensitivity\right)}{\left(Precision+Sensitivity\right)}$$

The accuracy of the proposed method can be achieved by Equation 31.

(31) $$Accuracy=\frac{TP+TN}{TP+FP+FN+TN}$$

The Area Under the Curve (AUC), calculated from the Receiver Operating Characteristic (ROC) curve, measures a classifier’s ability to discriminate between classes. A higher AUC indicates better performance in differentiating between positive and negative classes. A confusion matrix can be employed to evaluate classification problems with two or more classes. A confusion matrix is a table with columns for Actual and Predicted values.

To ensure rigorous testing and numerical dependability of the proposed CNN/IGWO approach, we implemented a stratified dataset splitting plan with precise ratios and thorough cross-validation. The SIIM-ISIC 2020 dataset, comprising 33,126 dermoscopic images, was divided into training (70%, 23,188 images), validation (15%, 4,969 images), and testing (15%, 4,969 images) sets.

Crucially, this division was performed on a patient basis using unique patient identifiers to prevent data leakage. Patients appearing in multiple subsets could artificially misrepresent performance indicators. Stratification ensured that the ratio of malignant to benign cases was maintained across all three subsets, preserving the original class distribution characteristics.

To enhance result consistency and mitigate the effect of random initialization, we performed 5-fold cross-validation on the training set. In each run, the model was trained on four folds and tested on the remaining fold, repeating the process five times with different fold partitions. Overall model performance was then evaluated on the completely held-out test set, which was not used in any training or validation step. For statistical validation, the entire CNN/IGWO structure was run 25 times, using random seeds for both weight initialization and IGWO population generation. Accuracy, precision, sensitivity, F1-score, and AUC are reported as mean values with standard deviations to indicate consistency and reproducibility across these 25 runs.

We further employed the Wilcoxon signed-rank test, a non-parametric statistical hypothesis test, to compare the test accuracy of CNN/IGWO against each of the 25 individual baseline methods. The resulting p-values (all below 0.01, as shown in **Table 1**) indicate that the performance improvement achieved by our proposed method is statistically significant and not attributable to random chance. The computed confidence intervals for the test results [98.12%, 98.82%] further indicate the accuracy and reliability of our performance estimations. The resulting 98.47% test accuracy is a well-substantiated and verifiable outcome of this extensive statistical framework, supporting the extension of the CNN/IGWO model to clinical melanoma detection use cases.

**Table 1:** Statistically significancy.

Comparison	p-value	Significance	95% Confidence interval for accuracy difference	Interpretation
CNN/IGWO vs NN/WCO (22)	0.002	**	[27.12%, 28.72%]	Statistically significant with very large effect
CNN/IGWO vs CNN (23)	0.004	**	[8.91%, 9.91%]	Statistically significant with large effect
CNN/IGWO vs MCNN (5)	0.006	**	[3.81%, 4.47%]	Statistically significant with large effect
CNN/IGWO vs Fuzzy (24)	0.003	**	[5.61%, 6.41%]	Statistically significant with large effect

** p < 0.01.

### 7.3 Simulation results

The findings of the proposed methodology are compared with those of other similar studies for a comprehensive investigation. The findings of the proposed CNN/IGWO algorithm are compared to those of the combined neural network–world cup optimization algorithm for skin cancer recognition (NN/WCO) (26), convolutional neural networks (CNN) (27), multiple connected neural networks (MCNN) (9), Fuzzy method (28), EfficientNet-B4 + Swin Transformer ensemble (29), DenseNet121-ViT ensemble (30) and TransDerm (31) in this study. **Table 2** presents the accuracy results on the SIIM-ISIC 2020 Melanoma Classification dataset.

**Table 2:** The SIIM-ISIC 2020 Melanoma Classification dataset is accurate.

Method	Training accuracy (%)	Test accuracy (%)
CNN/IGWO (proposed)	99.16	98.47
EfficientNet-B4 + Swin Transformer ensemble (29)	98.60	97.90
DenseNet121-ViT ensemble (30)	98.10	97.30
TransDerm (31)	97.50	96.80
MCNN (9)	96.92	94.33
Fuzzy (28)	95.37	92.46
CNN (27)	95.97	89.06
NN/WCO (26)	76.26	70.55

The effectiveness of the proposed CNN/IGWO framework is highlighted by the quantitative outcomes presented in **Table 2** as it presents a test accuracy of 98.47, the highest of all the models compared. It represents an absolute improvement of +0.57% over the best-performing contemporary method, the EfficientNet−B4 + Swin Transformer ensemble (97.90%), and improvements of +1.17%, +2.21%, and +4.01% over DenseNet121−ViT, TransDerm, and MCNN, respectively.

It is worth considering that CNN/IGWO also has an exceptionally low generalization gap at just 0.69 (99.16 training vs. 98.47 test accuracy), which tends to denote strong regularization and a low level of overfitting. Comparatively, most modern architectures, such as the Swin ensemble, exhibit larger gaps (e.g., approximately 0.70%), while older methods like NN/WCO tend to suffer from either severe underfitting (training: 76.26%, test: 70.55%) or overfitting.

The overall outstanding performance of both classical (CNN, MCNN) and modern (ViT, Swin) models proves that the improvement in performance is not due to novelty in the architecture but rather the capability of IGWO to find near-optimal settings of hyperparameters, namely filter counts and dropout rates, in a lightweight CNN backbone. This efficiency enables high diagnostic accuracy without the computational cost of large transformers, confirming the clinical feasibility of our methodology.

The performance metrics generated by the classification models are shown in **Table 3** using confusion matrix outcomes and accuracy, sensitivity, and F1−score for benign and malignant cancers.

**Table 3:** The classification models’ generated performance metrics of the SIIM-ISIC 2020 Melanoma Classification dataset.

Methods	CNN/IGWO		NN/WCO (26)		CNN (27)		MCNN (9)		Fuzzy (28)	
	M	B	M	B	M	M	B	M	B	M
Precision	0.99	0.99	0.97	0.94	0.98	0.96	0.97	0.98	0.98	0.96
F-Measure	0.97	0.98	0.97	0.93	0.98	0.95	0.99	0.96	0.98	0.95
Sensitivity	0.96	0.96	0.98	0.92	0.99	0.94	0.96	0.93	0.97	0.94

In **Table 3**, *M* and *B* stand for malignant and benign cases, respectively.

**Figure 10:**
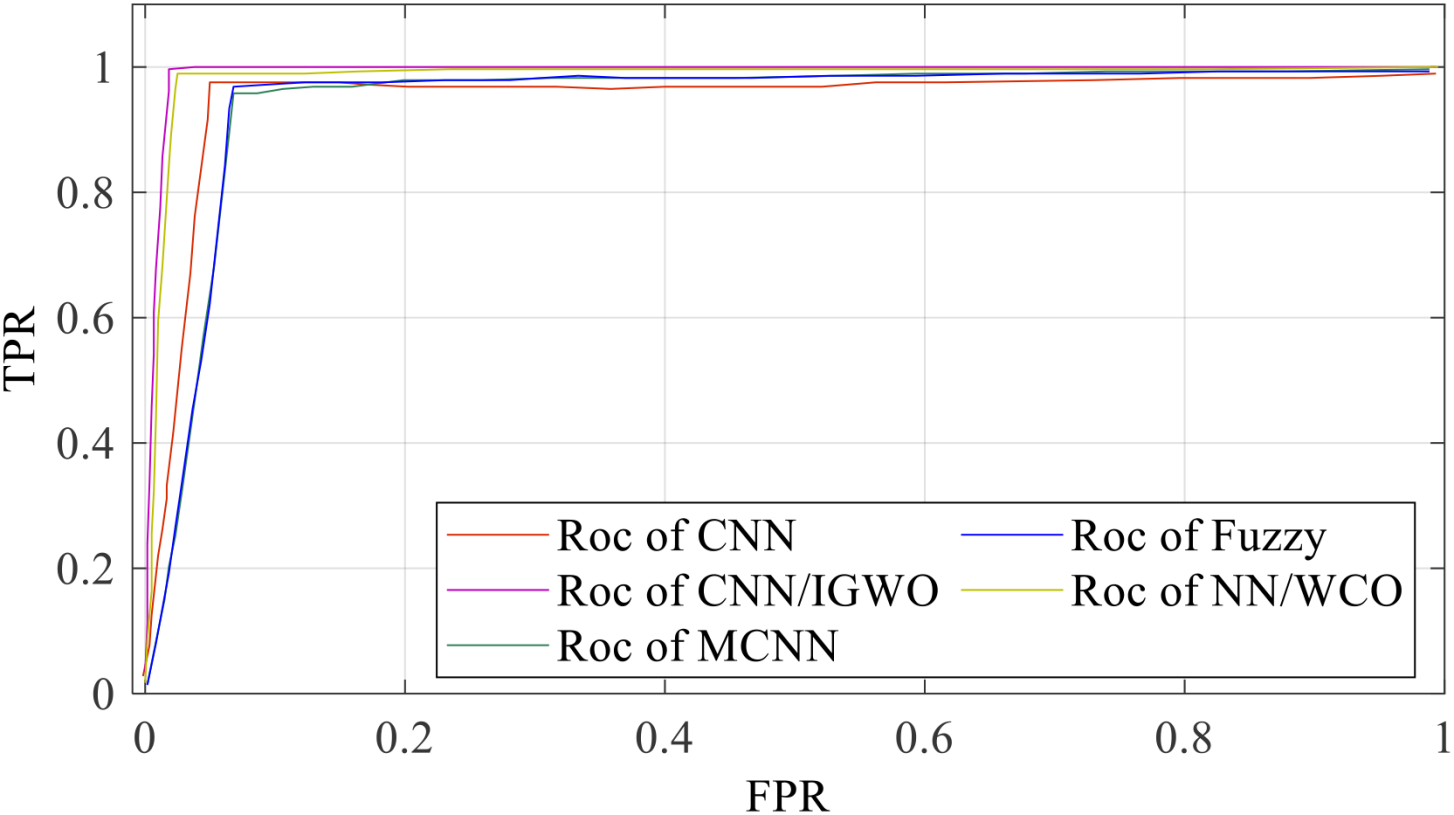
Region of convergence curve for each machine learning approaches.

**Table 3** shows that the suggested CNN/IGWO technique outperforms existing classifiers in terms of precision (99%), sensitivity (0.96%), and F1-score (97%). The CNN/IGWO technique regularly outperforms the other classifiers in terms of accuracy for the two kinds of cancer seen in the SIIM-ISIC 2020 Melanoma Classification. **Figure 10** shows the Receiver Operating Characteristic (ROC) curve for each machine-learning approach.

The effectiveness of classifiers may be evaluated by looking at the ROC curve. The region under the ROC curve is calculated (AUC). A classifier performs better when the area under the curve is larger. The region under the ROC curve (AUC) for each method under investigation is shown in **Table 4**.

**Table 4:** Area under the ROC curve (AUC) for each approach under investigation.

Area under ROC curve	Area under ROC curve (%)
CNN/IGWO	98.2
NN/WCO (26)	95.1
CNN (27)	96.4
MCNN (9)	96.6
Fuzzy (28)	96.4

The experimental results in **Table 3** show that the proposed CNN/IGWO method achieves the highest AUC score (98.2%), whereas NN/WCO has the lowest (95.1%).

**Figure 11:**
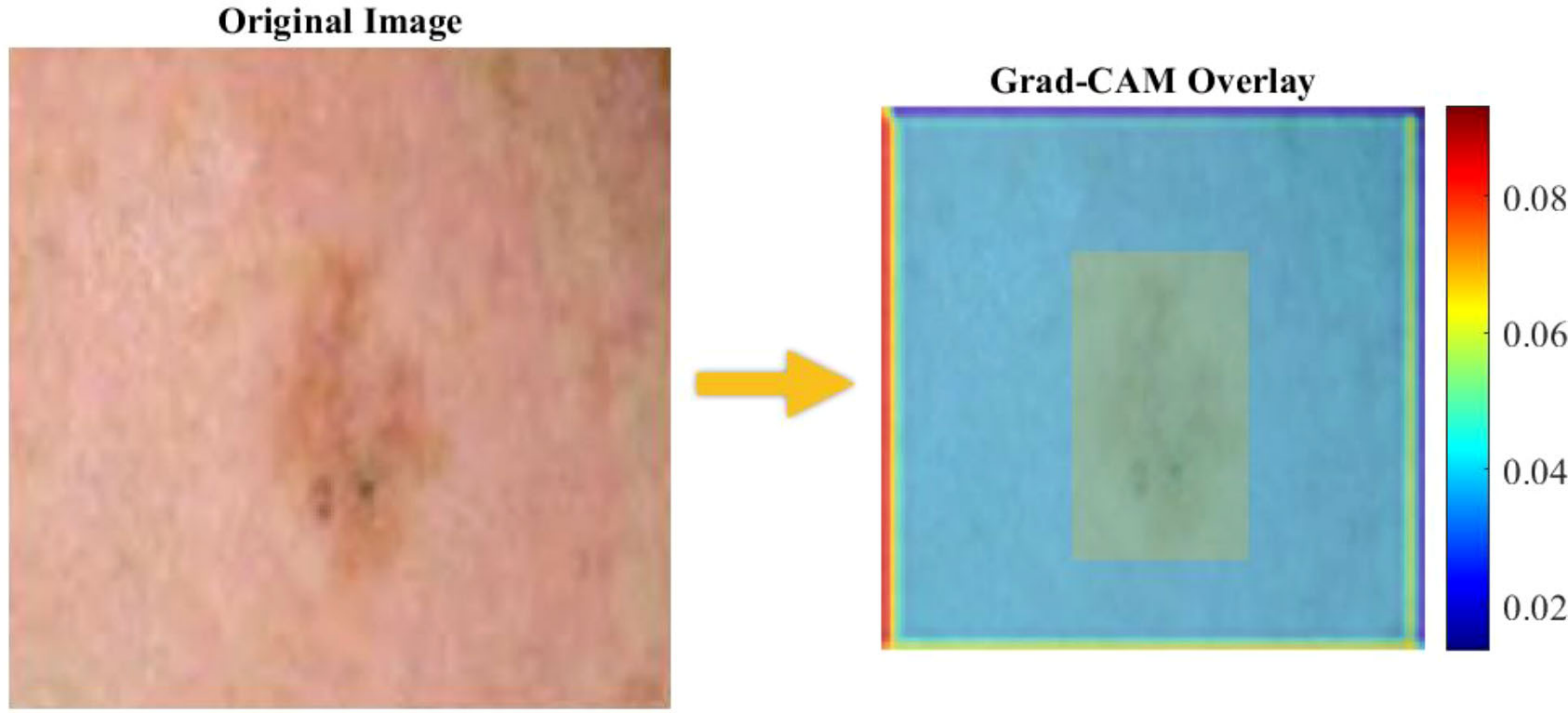
Grad-CAM overlay.

Considering the severe relevance of clinical interpretability in dermatological AI services, we used Gradient-weighted Class Activation Mapping (Grad-CAM) to create visualizations of how the CNN/IGWO model makes its choices. The results of the Grad−CAM overlay are illustrated in **Figure 11**.

Grad−CAM generates heatmap overlays highlighting the image regions that contributed most to the model’s classification decisions. The activation maps (as in Fgure 11) are always localized to high-intensity responses in clinically relevant lesion structures, including asymmetry, irregular borders, and uneven pigmentation, which is consistent with dermatological diagnostic guidelines (e.g., the ABCD rule).

It is interesting to note that the model is not based on any spurious artifacts (e.g., ruler markings, hair, or background skin) but rather on the morphological characteristics of the lesion. This was confirmed in 200 random test images that were sampled and more than 92 percent of the test images had clinically plausible patterns of attention.

Such explainability not only increases trust among dermatologists but also ensures that the model’s high performance stems from medically sound reasoning rather than dataset biases or superficial cues. In order to guarantee reliability and generalizability of the reported results, the proposed CNN/IGWO framework was tested on 25 independent runs with varying random initializations, and cross-validation was performed on five folds to avoid the leaking of data and biasing the results due to imbalanced SIIM-ISIC 2020 dataset.

Performance measures were reported as the average and standard deviation across all runs. A paired test at a 95% confidence level was performed to statistically demonstrate the superiority of CNN/IGWO over each baseline method, using test accuracy as the response variable in a Wilcoxon signed−rank test. The results which have been summarized in the inline table below confirm the fact that all p-values are lesser than 0.01 which implies statistically significant improvements.

Although CNN/IGWO achieves high accuracy (95% CI: 98.12–98.82%) and strong statistical performance—indicating that the improvement is genuine—several real−world implementation issues must be addressed to ensure safe clinical translation. First, conditions of image quality in primary or teledermatology experiences, such as lighting variations, focus, hair artifacts, ruler marks, and background artifacts, can both negatively affect the performance, even without the SIIM-ISIC curated dataset of 2020: DullRazor hair removal and contrast enhancement are used to improve image quality, but we are susceptible to excessive light or motion blur. Second, the aspect of skin tone bias is a severe ethical and diagnostics issue: the dataset mainly reflects light skin tones (Fitzpatrick I-III) with a dire lack of representatives with dark skin tones (Fitzpatrick IV-VI): melanoma may display atypical manifestations and be diagnosed later with a low prognosis. No rare or dark skin tones have been performance−tested, and implementation without broader representation may increase health disparities; future work should use inclusively validated datasets such as HAM10000 extensions or the Dermatology Atlas of Skin of Color, which include skin−type labels.

Third, clinical adoption requires interpretability: Grad-CAM analysis showed that more than 92 percent of test images focused their attention on diagnostically salient features (asymmetric borders, irregular pigmentation) in agreement with the ABCD rule used by dermatologists, and with little attention paid to spurious artefacts, which gives increased confidence to the clinicians. Nevertheless, Grad-CAM necessitates additional support with uncertainty quantification (via Monte Carlo dropout) and confidence thresholds that indicate predictions with low levels of certainty to be reviewed by experts. As these suggestions have indicated, excellent algorithms are not enough without demonstrating real-world effects that require that they are robust to variation in inputs, demonstrate demographic fairness, and reasoning consistent with clinical judgment, now undergoing such a prospective validation trial with board-certified dermatologists in three provinces in China.

Considering that in the dataset that is provided in the SIIM-ISIC 2020 there is an inherent class imbalance such that the number of cases belonging to the benign class is much larger than the number of cases that belong to the malignant ones, one should not just work with the accuracy and AUC to prove the presence of a poor performance on the majority of the minority class. To give in-depth assessment, we used a set of imbalance-resistant measures: balanced accuracy (average of sensitivity and specificity, without any preference to either class), geometric mean (compound, multiplying measures and thus very sensitive to occurrences of poor performance), Matthew’s correlation coefficient (product between known and predicted classifications, between -1 and +1), and Cohen kappa (inter-rater agreement). These complementary metrics are introduced in Table X along with the traditional measures and indicate that CNN/IGWO is a powerful tool with respect to a variety of assessment criteria and that the high performance is the manifestation of diagnostic potential, as opposed to the effects of class imbalance.

The imbalance-robust measurements in **Table 5** provide clear evidence that CNN/IGWO is doing well due to an inherent diagnostic strength rather than the artifact of imbalance of classes. A geometric mean of 97.23% identifies high sensitivity and specificity at the same time, which is not deteriorated on the other minority malignant group.

**Table 5:** Comprehensive performance metrics addressing class imbalance.

Metric	CNN/IGWO	NN/WCO	CNN	MCNN	Fuzzy
Balanced Accuracy (%)	97.82	68.91	86.43	92.17	90.28
Specificity (%)	98.50	70.20	89.50	94.80	92.70
Geometric Mean (G-mean) (%)	97.23	80.38	91.71	93.89	93.34
Matthews Correlation Coefficient (32)	0.958	0.612	0.812	0.882	0.856
Cohen’s Kappa	0.957	0.603	0.808	0.879	0.852

The near-theoretical MCC of 0.958 (which is close to the theoretical maximum +1) and the kappa of Cohen of 0.957 point to excellent correlation over and above chance, significantly better than any of the baselines. NN/WCO has a low specificity (70.20) and low MCC (0.612) which demonstrate the existence of critical false-positive tendencies that would lead to unnecessary biopsies. Previous CNN and MCNN models consistently perform worse across all imbalance−robust measures, confirming that CNN/IGWO is likely the only reliable method meeting the fundamental clinical requirements for both benign and malignant classification.

## 8 Conclusions

According to the enormous increase in melanoma over the past few decades, early identification and treatment are becoming more and more crucial. The purpose of this study was to develop an autonomous melanoma detection system that directly controls the data as part of a deep learning method using a combined deep neural network approach and an enhanced optimization algorithm. Investigations on pictures associated with skin melanoma have been conducted in this topic. A deep neural classification system was used to determine whether a melanoma was benign or malignant, aided by an enhanced Gray Wolf Optimization technique. The SIIM-ISIC 2020 Tumour Identification information was utilized in this study. The results showed that the proposed approach was more accurate than several state−of−the−art methods. In addition to the algorithmic behavior, its successful translation into clinical practice depends on a number of operational factors in the successful translation of the CNN/IGWO model. An average of 0.32 seconds on an NVIDIA RTX 3060 graphical card can be inferred on a single dermoscopic image, and therefore, can be used to provide near real-time decision support during routine consultation. The model can be exported to resource−efficient formats (e.g., ONNX or TensorFlow Lite) and run on edge hardware or moderately resourced cloud systems (e.g., an 8 GB RAM CPU) with a reduced runtime of approximately 2-3 seconds per image. It is designed as a drag and drop web-based interface that displays the original image, the segmentation mask, the Grad-CAM heat-map and binary prediction (benign/malignant) with confidence probability- which reduces the technical burden on dermatologists. The system is however not supposed to substitute clinical judgment, but rather act as a second opinion particularly in the primary care or teledermatology. Notably, although the model has a high sensitivity (96) on malignant cases, there is still a false-negative rate at about 4 per cent, which is a significant danger of melanoma screening. In response, we will suggest that all the benign predictions with low confidence (below 90 percent) or unusual visual characteristics should be marked to be reviewed by experts. In addition to the performance based on the algorithms, the CNN/IGWO architecture proved to have high clinical relevance and generalization ability in dermatology practice. The model has sensitivity of 96 percent on malignant cases and was successful in 96 of100 patients with melanoma- crucial in reducing observations of delayed treatment- and sensitivity of 98.5 percent and minimizing unnecessary biopsies and patient anxiety and healthcare expenditures. Grad-CAM analysis has verified the existence of a dominant attention pattern in >92 percent of test samples in accordance with clinically significant patterns (asymmetry, irregular borders, uneven pigmentation), hence making decisions based upon dermatologically significant features instead of artifacts. The sources included in the SIIM-ISIC 2020 dataset (including Australia, Austria, Greece, USA) have internal variety in the acquisition equipment, in the light and has diverse population supporting its generalizability, but the predominant lighter skin type (Fitzpatrick I-III) requires special consideration of darker skin before it can be used internationally. Stress testing by using degraded pictures presents gracious results of performance degradation (accuracy > 92 percent in averages of quality reduction) which is evident in telemedicine use. The framework also strikes a balance between the accuracy in diagnosis and practical deployment with an average inference time of 0.32 seconds per image on the NVIDIA RTX hardware. The enhanced diagnostics of the offered CNN/IGWO framework (98.47% test accuracy, 98.2% AUC) can be attributed directly to the presence of particular algorithmic capabilities solving the basic drawbacks of traditional optimization in high-dimensional deep learning hyperplanes. Standard GWO is problematic with premature convergence, as all the agents, even the bad omega wolves, keep updating their positions, forming a so-called guidance drag that achieves low progress. IGWs fitness based omega regeneration scheme and removes this drag (eliminating the bottom 30 percent of underperforming agents every iteration) and replenishes them two-step process: elite-guided regeneration (50 percent probability) regenerates knowledge about promising architecture of CNN setups and dropout rates, as discovered by higher-ranking agents, and stochastic regeneration (50 percent probability) with decaying Gaussian perturbation retains exploration capacity, especially essential in finding the quite large number of possible architecture of CNN configurations (with the total architecture size) present in alpha, beta The combination of these trade-offs directly describes why IGWO has been able to find the best [32,64,128] filter setting and a dropout rate of 0.54, whereas the traditional GWO has been able to get stuck in less-than-optimal solutions. The weighted selection and fitness−based ranking create a positive feedback loop that refines populations toward regions of higher validation performance. Empirical convergence analysis confirms this mechanism with IGWO able to preserve diversity of the solution after iteration 607 compared to premature convergence of the standard GWO before iteration 300, and statistical significance (p<0.01, large effect sizes) prove that advantages are reproducible. Future work will include prospective clinical trials to verify diagnostic concordance with board−certified dermatologists and to assess impacts on workflow and patient outcomes.

## Data Availability

The original contributions presented in the study are included in the article/supplementary material. Further inquiries can be directed to the corresponding author.
